# Transcriptional and Metabolic Insights into the Differential Physiological Responses of Arabidopsis to Optimal and Supraoptimal Atmospheric CO_2_

**DOI:** 10.1371/journal.pone.0043583

**Published:** 2012-08-20

**Authors:** Fatma Kaplan, Wei Zhao, Jeffrey T. Richards, Raymond M. Wheeler, Charles L. Guy, Lanfang H. Levine

**Affiliations:** 1 Center for Medical, Agricultural and Veterinary Entomology, Agricultural Research Service, United States Department of Agriculture (USDA-ARS), Gainesville, Florida, United States of America; 2 MedImmune LLC, Gaithersburg, Maryland, United States of America; 3 Enterprise Advisory Services Inc., QinetiQ North America for Engineering Services Contract (ESC), Sustainable Systems Applied Research, Kennedy Space Center, Florida, United States of America; 4 NASA Engineering Directorate, Kennedy Space Center, Florida, United States of America; 5 Plant Molecular and Cellular Biology Program, Department of Environmental Horticulture, University of Florida, Gainesville, Florida, United States of America; Max Planck Institute for Chemical Ecology, Germany

## Abstract

**Background:**

In tightly closed human habitats such as space stations, locations near volcano vents and closed culture vessels, atmospheric CO_2_ concentration may be 10 to 20 times greater than Earth’s current ambient levels. It is known that super-elevated (SE) CO_2_ (>1,200 µmol mol^−1^) induces physiological responses different from that of moderately elevated CO_2_ (up to 1,200 µmol mol^−1^), but little is known about the molecular responses of plants to supra-optimal [CO_2_].

**Methodology/Principal Findings:**

To understand the underlying molecular causes for differential physiological responses, metabolite and transcript profiles were analyzed in aerial tissue of Arabidopsis plants, which were grown under ambient atmospheric CO_2_ (400 µmol mol^−1^), elevated CO_2_ (1,200 µmol mol^−1^) and SE CO_2_ (4,000 µmol mol^−1^), at two developmental stages early and late vegetative stage. Transcript and metabolite profiling revealed very different responses to elevated versus SE [CO_2_]. The transcript profiles of SE CO_2_ treated plants were closer to that of the control. Development stage had a clear effect on plant molecular response to elevated and SE [CO_2_]. Photosynthetic acclimation in terms of down-regulation of photosynthetic gene expression was observed in response to elevated [CO_2_], but not that of SE [CO_2_] providing the first molecular evidence that there appears to be a fundamental disparity in the way plants respond to elevated and SE [CO_2_]. Although starch accumulation was induced by both elevated and SE [CO_2_], the increase was less at the late vegetative stage and accompanied by higher soluble sugar content suggesting an increased starch breakdown to meet sink strength resulting from the rapid growth demand. Furthermore, many of the elevated and SE CO_2_-responsive genes found in the present study are also regulated by plant hormone and stress.

**Conclusions/Significance:**

This study provides new insights into plant acclimation to elevated and SE [CO_2_] during development and how this relates to stress, sugar and hormone signaling.

## Introduction

The influence of increasing atmospheric [CO_2_] on plant physiology (photosynthesis, respiration, and stomatal conductance) has been studied across many species [1], [2]. In C3 species, enriching [CO_2_] up to 3 times the current ambient level generally stimulates photosynthesis and reduces stomatal conductance [3], [4], [5], [6]; but the response of respiration to elevated CO_2_ is more variable and uncertain [1], [7]. Long-term exposure to elevated [CO_2_] leads to a reduced stimulation of net CO_2_ uptake, which is interpreted as photosynthetic acclimation due to in part reduced amount of active RuBisCO [8]. More specifically photosynthetic acclimation is defined as a decrease in the maximum carboxylation rate *V*_c,max_ of Rubisco and maximum electron transport leading to RuBP regeneration *J*_max_ [9]. Increasing [CO_2_] beyond the photosynthetic saturation point should provide no further benefit to plants, and extremely high or super-elevated (SE) [CO_2_] (≥4,000 µmol mol^−1^) could result in a variety of negative effects on plants. In fact, SE [CO_2_] has been shown to cause leaf injury [10], [11], [12], [13], foliar deformation [14], reduced photosynthesis and respiration rates, lower seed yields, and decreased total biomass accumulation [12], [15]. Furthermore, SE [CO_2_] can impair stomatal closure [16] and interfere with abscisic acid mediated stomatal controls [17], which could potentially reduce plants’ tolerance to water stress and environmental pollutants. In summary, plants respond to elevated and SE CO_2_ very differently.

Understanding the mechanism by which plants respond to moderately elevated CO_2_ and SE CO_2_ has practical implications for plant adaptation to climate change, survival in extreme environments as well as preservation of plant functions (water and atmospheric gas cycles) as an essential component of the bioregenerative system. Although there have been many efforts to reveal the molecular basis for the response to elevated CO_2_ [7], [18], [19], [20], [21], [22] using transcriptome profiling, none have focused on plants exposed to SE CO_2_. Studies on the metabolic consequences of [CO_2_] enrichment are even more limited [18], [22], [23], [24], with the emphasis frequently placed on carbohydrate metabolism [25], or a small group of compounds from specific biosynthetic pathways such as flavonoids or glucosinolates [26], [27].

Several studies have focused attention on the effects of elevated [CO_2_] and growth environment on gene expression and metabolism [24], [28], [29], [30], [31]. Gene expression levels for plants grown in growth chambers were compared with those for plants grown in Free Air Concentration Enrichment (FACE) rings. The greatest number of changes in gene expression was observed between growth chamber and ambient field conditions. Two to four times the number of transcripts were either up- or down-regulated in controlled environment versus field ambient conditions compared with high versus low [CO_2_] [28]. In contrast, the abundance of fewer than 50 transcripts in poplar differed significantly between different [CO_2_] environments [30], while as much as 8% of the Arabidopsis transcriptome was found to be responsive to elevated [CO_2_] [32]. The identity of many of the [CO_2_]-responsive genes suggests elevated [CO_2_] stimulates the respiratory breakdown of carbohydrates, which provides increased energy and biochemical precursors for leaf expansion and growth at elevated [CO_2_] [31]. Increased availability of [CO_2_] also resulted in a decreased expression of transcripts for components of the light harvesting and Calvin cycle machineries [33]. Yet, a number of [CO_2_] responsive genes with increased transcript abundance were for functions that distribute carbon. The response to distribute carbon led to changes in the ratios or balances between hexoses/sugars, organic acids and amino acids under elevated [CO_2_] [33]. In some studies, metabolite responses were observed to parallel gene expression patterns [32]. In others, metabolites responses and transcriptional responses differed [22].

Photoassimilate pool sizes in Arabidopsis become enhanced in high [CO_2_] in an ecotype-specific manner, while at the same time, elevated [CO_2_] stimulates short-term growth and carbon gain independent of down-regulation of plastid functions. However, altered expression of genes involved in nitrogen metabolism was concluded to resemble patterns observed under N-deficiency [24]. Overall, carbon fixation with a smaller commitment of resources in elevated [CO_2_] appeared beneficial, with the extra C only partially utilized possibly due to disturbance of the C:N ratio. Different ecotypes perceived elevated [CO_2_] as a metabolic perturbation that necessitated increased functions consuming or storing photoassimilate. Also in Arabidopsis, elevated [CO_2_] favored adjustments in reactive oxygen species (ROS) homeostasis and signaling that defined genotypic markers [24].

In spite of several decades of effort on elevated [CO_2_]-related research, understanding the mechanisms by which plants respond and acclimate to elevated [CO_2_] remains incomplete. Little is known about the direct linkages of gene expression (GE) and metabolism on a genome to metabolome-wide scale [34]. The emergence of non-targeted metabolite profiling (MP) or wide-scale metabolomic analyses offers the opportunity to reveal functional linkages between gene expression patterns and changes in metabolite steady-state levels [35], [36], [37]. In light of the fact that regulatory control is exerted at multiple levels from genes, proteins to metabolites, neither stand-alone transcriptomic or proteomic studies can provide a clear picture of how GE translates into metabolic consequences. In this study, we investigated the consequences of elevated and SE [CO_2_] at the transcript and metabolite level in Arabidopsis grown at near-saturating light levels.

## Results

### Elevated and Super Elevated [CO_2_] Results in Different Physiological Responses

**Figure 1:**
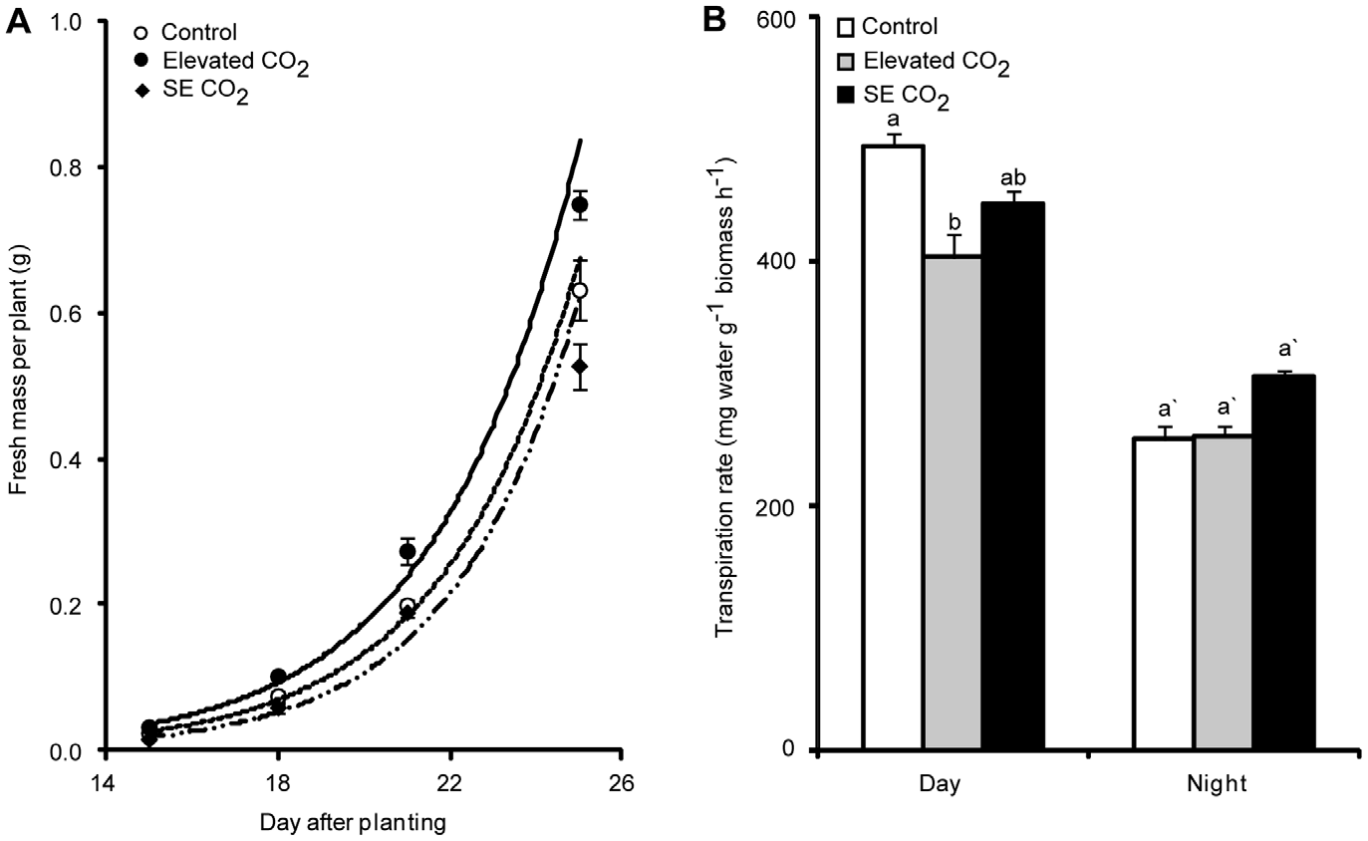
Effect of atmospheric [CO_2_] on biomass accumulation and transpiration of Arabidopsis. A) For biomass accumulation fresh weight is measured. Three experiments were done for each time point. Each experiment had 3 contained pots which had 6 plants for fresh weight measurements. B) Transpiration rate during the day and at night. Three experiments were done for each data point and each data point had 3 measurements.

The physiological responses of Arabidopsis to elevated and super-elevated CO_2_ differ significantly (Figure 1). Biomass accumulation rate was enhanced by elevated CO_2_, but not by super elevated (SE) CO_2_. Instead, a slight decrease was observed in biomass by SE CO_2_ (Figure 1A). Furthermore, plant transpiration rate during the day was significantly reduced by elevated CO_2_, but unaffected by super elevated CO_2_. Transpiration rate was restored to the level of ambient CO_2_-grown plants at night (Figure 1B). Although SE CO_2_ reduced plant biomass, it positively affected the progression of plant development, which was closer to that of elevated CO_2_ (Table 1). It took 4 days to progress from early vegetative stage (1.09 leaf stage) to late vegetative stage (1.14 leaf stage, right before reproductive stage) for both 1,200 and 4,000 µmol mol^−1^ grown plants, but 7 days for plants grown in ambient CO_2_ suggesting that plant development was accelerated by the enrichment of [CO_2_] to 1,200 and 4,000 µmol mol^−1^ in the atmosphere. The differential growth and development rate induced by increasing atmospheric [CO_2_] was taken in to account for sampling for the transcript and metabolite profiling and sampling time was standardized based on the actual developmental stage instead of the apparent age, day after planting.

**Table 1:** Plant Growth Conditions (Average ± standard deviation) and Development.

[CO_2_], µmol mol^−1^	RH, %	Temperature, °C	DAP* to 1.09 Stage	DAP to 1.14 Stage
400±18	66±0.6	20.1±0.00	18	25
1200±68	66±0.6	20.1±0.01	17	21
4000±416	67±0.5	20.1±0.01	17	21

* DAP: Day after planting.

### Differential Response to Elevated and SE CO_2_ at the Molecular Level is Developmentally Dependent

**Figure 2:**
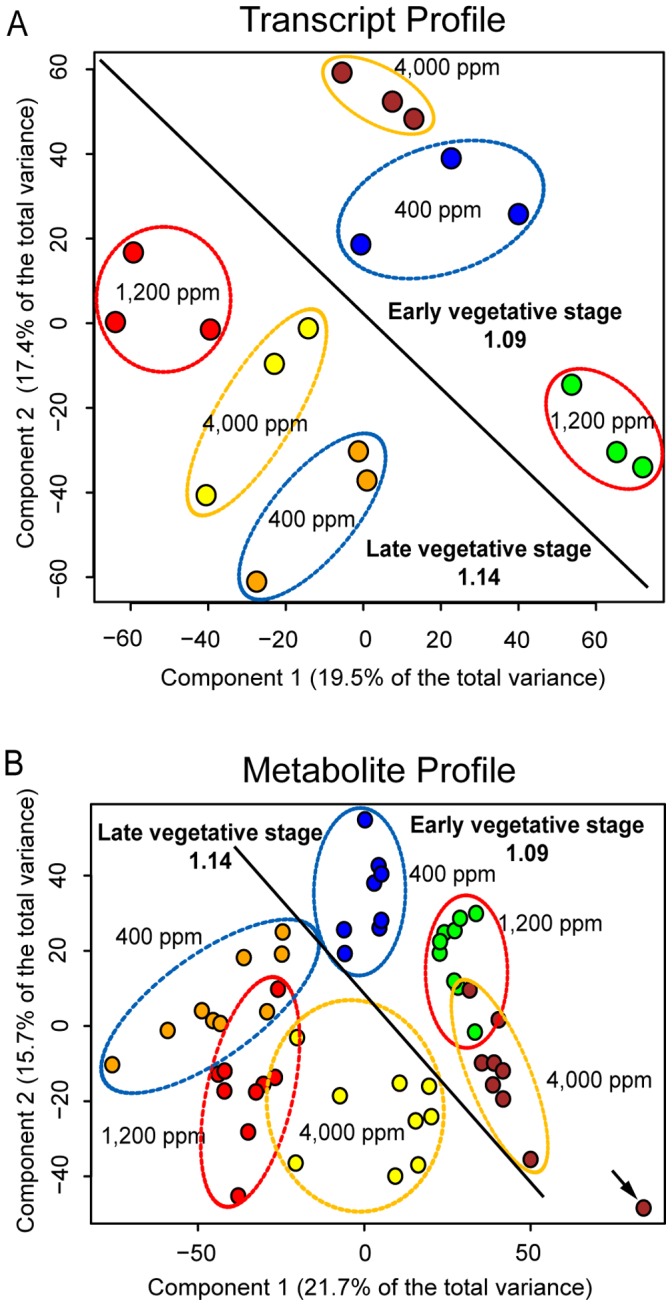
The effect of [CO_2_] and developmental stage on global transcript profile and metabolite profiles for low molecular weight polar compounds. **A**) PCA of transcript profile (n = 3 biological experiments). Each colored-coded point represents independent biological samples. The numbers 1.09 and 1.14 indicate the developmental stage as defined in the Growth Stage Model [63]. The entire dataset of 12106 (represented 11,418 unique mRNAs) probe sets present was used for this analysis. **B**) PCA analysis of metabolite profiling data (n = 3 biological replicates x3 analytical replicates). Each biological experiment was a pool of 12–16 plants and the same pool of ground tissue was used for both RNA extraction and metabolite extraction. The arrow points to an outlier in the metabolite profiling data set.

Principal component analysis (PCA), which reduces multivariate data complexity to identify patterns in large data sets, was performed to assess overall experimental variation and test for the presence of differences between elevated and SE CO_2_ treatment in transcript and metabolite profiling data. Analysis of the expression of 11,418 unique mRNAs revealed that four major principal components (PC) accounted for 54% of the total variance suggesting that the majority of the variance was due to the treatments. The first two components accounted for 37% of the total variance and differentiated among [CO_2_] treatments as well as developmental stages of the same CO_2_ treatment (Figure 2A). At both early and late vegetative developmental stages, the variance patterns of transcript profiles in 1,200 µmol mol^−1^-grown plants were very distinct from that of the control plants (400 µmol mol^−1^) and from that of SE (4000 µmol mol^−1^) CO_2_-grown plants. Surprisingly, the SE CO_2_ profiles indicated a move of the transcriptional activity back closer to the control state (same PC1, different PC2) at both early and late vegetative stage. This is consistent with the trend of biomass accumulation and water transpiration data (Figure 1) where values from the super elevated CO_2_ was closer to those at the ambient CO_2._ However, the observed transcriptional activity in PCA could not have been predicted from physiological data, because components of PCA (Figure 2) represent many transcripts that regulate not only plant growth and stomatal control (thus water transpiration), but also other biochemical and physiological processes. For instance, SE [CO_2_] has been shown to cause leaf injury [10], [11], [12], [13], foliar deformation [13], reduced photosynthesis and respiration rates, lower seed yields, and decreased total biomass accumulation [12], [15], we expected a fairly distinct transcriptional activity away from both control and elevated CO_2_. Examination of PCA components revealed that variance contributing transcripts are involved in photosynthesis, carbohydrate metabolism, glycolysis/gluconeogenesis, fermentation, TCA cycle, mitochondrial electron transport, ATP synthesis, cell wall, lipid metabolism, amino acid metabolism, secondary metabolism, hormone metabolism, stress responses, redox, polyamine metabolism, signaling, development, transport, and others encoding proteins with unknown function.

Low molecular weight metabolites were extracted from the same pool of tissue that was used for transcript profiling. The metabolite profiling (168 metabolite and mass spectral tags) results were also analyzed by PCA to determine the overall variance in the dataset and variance within individual experiments. Analysis of the metabolite profiles revealed three major components which accounted for 50% of the total variance in the dataset. Consistent with the transcript profile, the first two PCs accounted for 37.4% of the total variance and were able to differentiate [CO_2_] treatment and developmental stage effects (Figure 2B). While the effect of CO_2_ treatment and developmental stage on these metabolites was evident, there was some degree of overlap of the clusters for [CO_2_] treatments, particularly in the late vegetative stage. In contrast to transcript profile, metabolic profile of SE CO_2_-grown plants were away from the control and close to that of elevated CO_2_-grown plants at early vegetative stage. The first PC consists of mainly amino acids such as Glu, Ala, Gln and pyroglutamic acid and organic acids including some of the TCA cycle intermediates (citrate and fumarate). The second PC is made of mainly sugars like sucrose, glucose and fructose and photorespiratory intermediates (glyceric acid, Ser, Gly). All the metabolites in 1^st^ and 2^nd^ components appear to be associated with C/N balance. The third PC is mainly composed of compounds of mostly unknown identity. In summary, PCA showed that most of the total variance in the transcript and metabolite profile data sets was due to CO_2_ treatment and development. Both data sets suggested that there is an interaction between development and CO_2_ response and this interaction was much more evident in the transcript profile data set (Figure 2).

**Figure 3:**
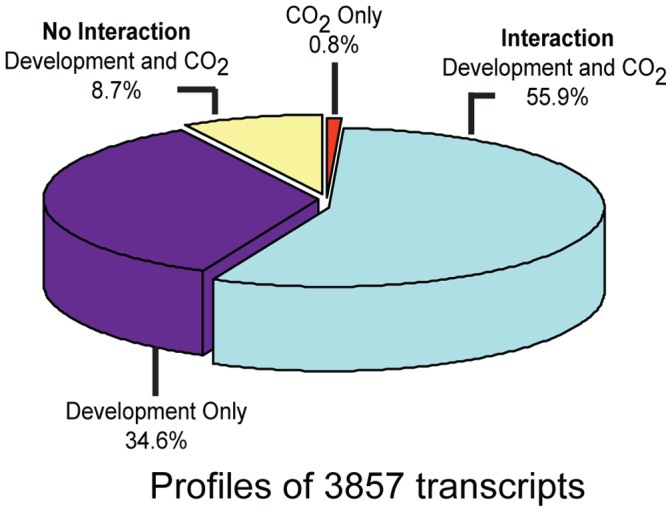
The effect of [CO_2_] and developmental stage on transcript profile. A mixed model effect analysis on transcript profile data revealed that the signal intensities of 3,857 transcripts showed significant changes at p<0.001 as a function of [CO_2_], developmental stage, and the interaction between [CO_2_] and developmental stage.

In order to differentiate the regulation due to development, CO_2_ exposure and interaction of development and CO_2_ exposure, the transcript profile data were analyzed using a mixed effect model (nlme package in R software). The signal intensities of 3,857 transcripts were found to be statistically significant (p<0.001) as a function of [CO_2_], developmental stage, and the interaction between [CO_2_] and developmental stage (Figure 3). Surprisingly, 0.8% of the transcripts were regulated by only CO_2_, 8.7% by both development and [CO_2_] independently and ∼56% of the transcripts were regulated through interaction of development stage and [CO_2_] (Figure 3, Table S1A and B) suggesting that development modulates how plants respond to CO_2_. Table S1A and B show only the level of transcripts that changed 2 times or more. These data revealed that Arabidopsis responds to elevated and SE CO_2_ differentially in a development dependent manner at the transcriptional level for many pathways including photosynthesis.

**Figure 4:**
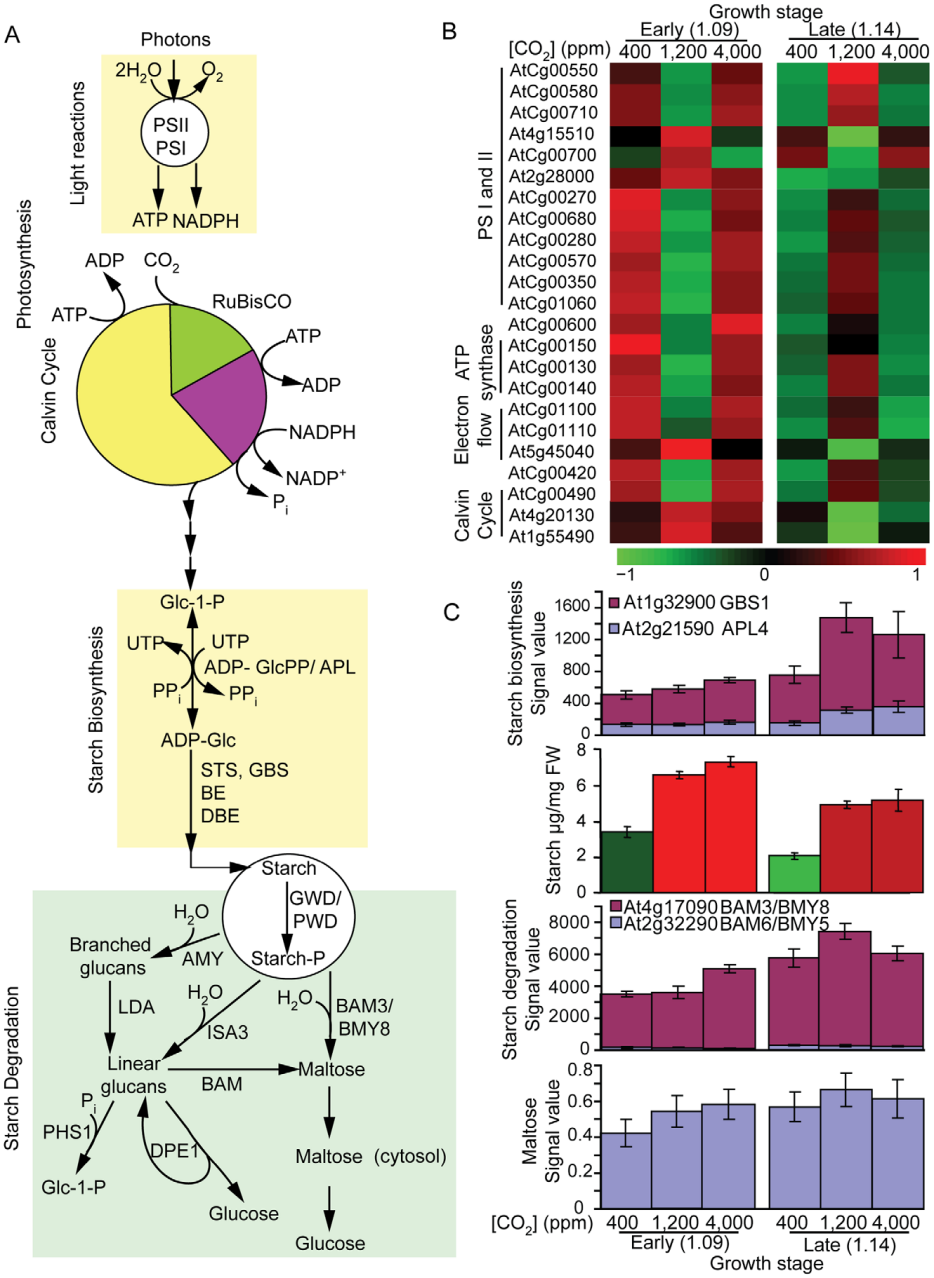
Influence of [CO_2_] and development on photosynthesis and starch metabolism. **A**) Photosynthesis and starch biosynthesis and degradation pathways. **B**) Heat maps of the transcript profiles of photosynthesis. For each gene, its expression level was normalized across all samples so that the mean expression is 0 and the variance is 1. Higher levels are represented with color red of increasing intensity, and lower levels are represented with green of increasing intensity. The increased or reduced expression is relative to the mean expression. All transcript levels shown was statistically significant (p-value ≤0.001) with 2 fold or more increase or decrease at any one treatment. **C**) Starch biosynthesis, starch content, starch degradation and maltose content (n = 3 biological replicates ×3 analytical replicates). Starch levels were significantly higher in elevated and SE [CO_2_] treatments (students t-test p-value <0.05) than the control.

**Figure 5:**
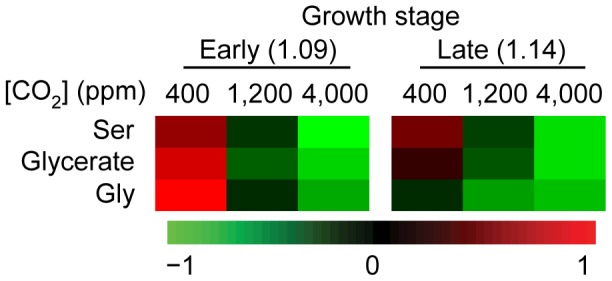
Heat map of the profiles of metabolites involved in photorespiration. All changes in metabolite levels had a statistically significant response (p-value ≤0.001) and more than 2 fold increase or decrease at any one treatment.

**Figure 6:**
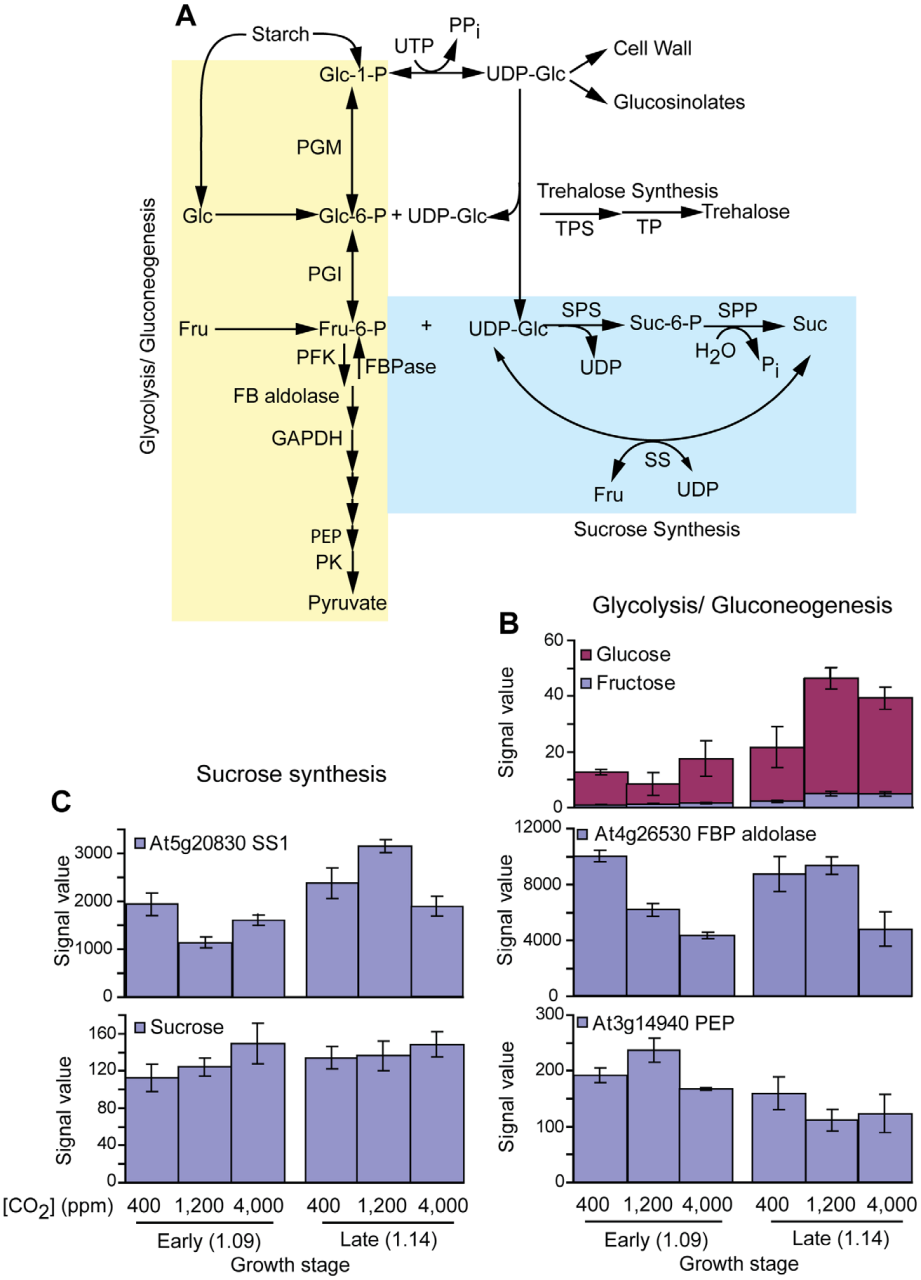
Glycolysis/gluconeogenesis, trehalose and sucrose biosynthesis. **A**) Glycolysis/gluconeogenesis, trehalose and sucrose biosynthetic pathways. **B**) Transcript and metabolite profiles of glycolysis/gluconeogenesis. **C**) Transcript and metabolite profiles of sucrose biosynthetic pathways. All transcript and metabolite levels shown had a statistically significant response (p-value ≤0.001) and more than 2 fold increase or decrease during any treatment.

Both elevated and SE [CO_2_] increased photosynthesis as indicated by increased starch content. The starch content was tightly associated with transcript levels of starch biosynthetic genes and degradation genes (Figure 4), photorespiratory metabolites pools (Figure 5), soluble sugar pools (Figure 6), but not photosynthetic genes involved in light cycle and Calvin Cycle (Figure 4). CO_2_ enrichment to 1,200 µmol mol^−1^ resulted in a reduced abundance of transcripts of the PS I and II genes (9 out of 11) at the early vegetative stage (1.09 leaf stage), but enhanced abundance of the same transcripts at the late vegetative stage (1.14 leaf stage), suggesting the influence of CO_2_ on photosystem I and II transcripts is dynamic and likely development-dependent (Figure 4B). In contrast, the abundance of these transcripts in plants grown under a CO_2_ concentration of 4,000 µmol mol^−1^ (Figure 4B, Table S1B) was unchanged relative to those in plants grown under ambient CO_2_ of 400 µmol mol^−1^ at either early or late vegetative stage. Furthermore, the transcript level of the gene (AtCg00490) for RuBisCO, the enzyme that catalyzes the first step in Calvin Cycle, showed a very similar pattern to those for the PSI and II. In contrast, transcript levels of the genes (At4g20130 and At1g55490) for RuBisCO interacting proteins showed an opposite response. These data revealed that Arabidopsis responds to elevated and SE CO_2_ differentially at the transcriptional level for genes for photosynthesis.

Increasing atmospheric [CO_2_] not only affects starch synthesis, but also its degradation at the transcriptional level (Figure 4C). Furthermore, the influence of CO_2_ concentration on transient starch content is development dependent (Figure 4C). Elevated CO_2_ enhanced transcript levels of the genes that encode for starch biosynthesis enzymes, glucose-1-phosphate adenyl transferase (APL4) that catalyzes the first and rate limiting step and granule bound plastidic starch synthase (GBS1) at both early and late vegetative stages. The CO_2_-induced increase in transcripts for starch synthesis was more pronounced at the late vegetative stage than at the early vegetative stage. In contrast, mid-day starch content increased significantly by both [CO_2_] (1,200 and 4,000 µmol mol^−1^) with no difference between the two CO_2_ treatments (students’ t-test p<0.05) and the increase in starch content was more at the early vegetative stage. This result suggested increased starch degradation at the late vegetative stage. Concomitantly, the transcript levels of genes encoding starch degradation enzymes such as beta-amylase (BAM3/BMY8, chloroplast localized) were higher at the late vegetative stage than early vegetative stage for each respective CO_2_ treatment, and increased in response to SE [CO_2_] at early vegetative stage and elevated [CO_2_] at late vegetative stage. The content of maltose, a starch degradation product, echoed the response of BAM3/BMY8 to increasing CO_2_ and developmental stage. At any given CO_2_ concentration, late vegetative stage had lower starch than younger plants. There was a positive correlation between the starch content at the early vegetative stage and the rate of leaf development to the late vegetative stage suggesting that a higher mid-day starch content was associated with a faster rate of development.

Starch degradation can contribute to pools of free glucose and fructose (Figure 6A), which are precursors of hexose phosphates (Glc-1-P, Glc-6-P and Fru-6-P). Consistent with the reduced starch content in the late vegetative stage, free glucose and fructose content increased more than 2 fold at the late vegetative stage (Figure 6B). This increase is more pronounced in the elevated than SE CO_2_–grown plants (Figure 6B). Surprisingly, changes in free glucose and fructose levels were not reflected by the relative abundance Glc-6-P and Fru-6-P. This could be because hexose phosphates (Glc-1-P, Glc-6-P and Fru-6-P) are intermediates and precursors for many biosynthetic pathways (Figure 6A) including sucrose, trehalose, and cellulose biosynthesis. Because of their central role as precursors for many biosynthetic pathways, their relationship to downstream pathways like sucrose and trehalose biosynthesis is difficult to resolve. For example, Glc-6-P and Fru-6-P content showed a small, less than 2 fold, but significant (p<0.001) increase to elevated and SE [CO_2_] at the early vegetative stage, but not at the late vegetative stage (data not shown). While sucrose signal levels showed a very small but significant increase, less than 1.5 fold, as [CO_2_] increased at both developmental stages (Figure 6C). The sucrose synthase (SS1) transcript profile was different than the sucrose content profile (Figure 6C). At the early vegetative stage, SS1 transcript levels decreased in response to [CO_2_] treatment. This was more pronounced with the 1,200 µmol mol^−1^ [CO_2_] treatment. At the late vegetative stage, SS1 transcript levels increased, in response to elevated [CO_2_] and decreased in response to SE [CO_2_]. Overall, individual soluble sugar levels were different but the total soluble sugars (trehalose, maltose, sucrose, glucose and fructose) were higher at the early vegetative stage in response SE [CO_2_] and at the late vegetative stage in response to both elevated and SE [CO_2_]. This suggests that starch degradation might be contributing the increase in soluble sugar levels during late vegetative stage.

RuBisCO catalyzes the first step in both photosynthesis, where CO_2_ is assimilated, and photorespiration, where O_2_ is assimilated leading to net loss of carbon and slow plant growth. Similar to RuBisCO transcript profile, changes in the levels of the other transcripts of genes involved in photorespiration were very subtle (data not shown), less than 2 fold, in response to both elevated and SE CO_2_. Furthermore, steady-state levels of metabolites associated with photorespiration, glycine, serine and glycerate, exhibited a similar response pattern to the two CO_2_ enrichments, i.e. decreased as [CO_2_] increases regardless of the developmental stages examined (Figure 5, Table S2). The decrease in photorespiratory metabolites was consistent with increase in CO_2_ enrichments and starch accumulation.

### Elevated and SE CO_2_ Interaction with Sugar and Plant Stress Signaling

**Figure 7:**
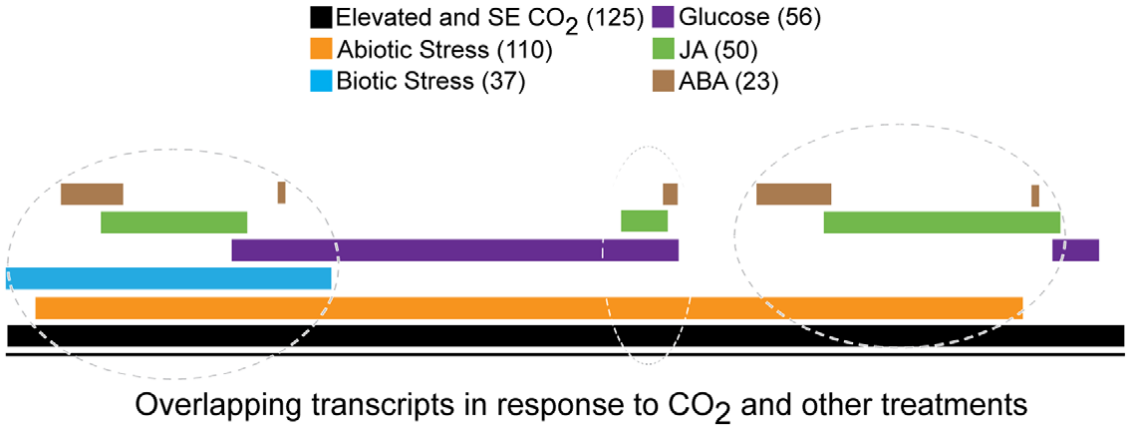
Interaction of CO_2_ adaption with sugar, hormone signaling and stress. The 559 CO_2_ regulated transcripts (p<0.001) in the present study were compared to publicly available microarray data. The number of transcripts that overlap is shown on the figure. There are group of CO_2_ regulated genes are also regulated by biotic, abiotic, sugar, JA and ABA. There are another group CO_2_ regulated genes are also regulated by sugar and abiotic stress. Biotic stress is for *Phytophthora infestans* and *Botrytis cinerea*. Abiotic stress is for cold, osmotic and salt stress.

Because soluble sugar levels were increased by growth in elevated and SE CO_2_ and soluble sugars have been known to accumulate during stress and regulate genes expression, we compared 559 CO_2_-responsive transcripts in Table S1B with published microarray data for glucose-regulated genes [38]. Out of the 559 CO_2_ responsive transcripts, 56 transcripts were regulated by glucose (Figure 7, Table S3). None of the glucose regulated (56 genes) in our data set were related to photosynthesis consistent with Ludewig and Sonnewald’s [21] findings, but many of them are regulated by abiotic stresses.

Abiotic stress results in accumulation of soluble sugars [39], [40] and is known to induce plant defense related hormones [39]. In order to understand the interaction of elevated CO_2_ and stress, CO_2_-responsive transcripts (Table S1B) were examined using *The Bio-Array resources for Arabidopsis Functional Genomics* for abiotic stress (temperature stress, salt, drought, oxidative, osmotic, UV-B, genotoxic stress), and pathogen responses (*Phytophthora infestans, Botrytis cinerea, Pseudomonas syringea, Erysiphe orontii*, and elicitors derived from bacteria and oomycetes). Out of 520 transcripts analyzed (Figure 7), the majority of responses (110 transcripts) were linked to abiotic stress (cold, osmotic and salt stress), and a smaller group (37 transcripts) was associated with biotic stress (*Phytophthora infestans,* and *Botrytis cinerea*). Additionally, many of the abiotic and biotic stress regulated transcripts have been shown to be regulated by plant hormones and sugars.

Involvement of jasmonic acid (JA) and abscisic (ABA) in biotic and abiotic stress responses is well known [41], [42]. To further explore the interaction of elevated CO_2_ and stress related phytohormones, 520 of the 559 CO_2_-responsive transcripts (Table S1B) were examined using *The Bio-Array Resources for Arabidopsis Functional Genomics* and *Transcriptional Regulation by ABA Signaling (TRABAS)* [43]. In both databases, a majority of the hormone-regulated genes were JA and ABA regulated (Figure 7), and a small group of genes were brassinolide (BR)-regulated. For that reason, we focused on JA, ABA, and BR-regulated genes and compared our dataset to published microarray results [43], [44], [45], [46], [47]. Out of the 559 transcripts, 50 appear to be regulated by JA, 23 regulated by ABA (Figure 7, Table S3) and 20 regulated by BR (Table S3). Examples of these transcripts include ATHB-7 (At2g46680) Homoebox 7 transcription factor, APL4 (At2g21590), the enzyme that catalyzes the first step in starch synthesis, LPT3 (lipid transfer protein 3 (At5g59320), CORI3 (coronatine induced, or jasmonic acid responsive) (At4g23600). Other transcripts known to be responsive to JA or ABA or both hormones included GCN5-related N-acetyltransferase (GNAT) family protein (At2g39030) jacalin lectin family proteins, myrosinases jacalin lectin, pathogenesis-related proteins (PR4), LPT4 (lipid transfer protein 4), alcohol dehydrogenases, ATCLH1 (coronatine induced protein1), NRT1 (nitrate transporter 1), ATHB-12 (homeobox protein), RD26, RAB18, and LTI 30. Some of the brassinolide-regulated transcripts were ATTI1 (trypsin inhibitor protein 1), auxin responsive protein, pectinesterase family protein, and ARK3 (receptor kinase), ACD6 (accelerated cell death 6) and NIA1 (nitrate reductase 1) (Table S3). These data suggest that elevated or SE [CO_2_] induces abiotic stress related responses. This is consistent with findings of recent study [22], where they observed differential regulation of transcripts in response to elevated CO_2_ and salt stress, and these transcripts included lipid transfer proteins, GCN5-related N-acetyltransferase (GNAT) family protein, low temperature and salt responsive protein, pectinesterase family proteins and pathogenesis-related proteins. A subset of common responses to high CO_2_ and abiotic stress may be regulated through JA, ABA, glucose signaling and/or a cross talk between hormone and sugar signaling which may play a role during adaptation to elevated and SE CO_2_ in a development-dependent manner.

## Discussion

It is well known that plants respond to elevated [CO_2_] at physiological, metabolic and molecular levels [6], [28], [29], [30], [32]. Transcriptome or proteome profiles by themselves cannot predict how gene expression translates into metabolic consequences because regulatory control is exerted at multiple levels from genes, proteins to metabolites. Our experimental design enabled us to distinguish the effects of the innate developmental program from that of increased [CO_2_] on gene expression and metabolite profiles by sampling plants at the same developmental stage instead of the days-after-planting (DAP). Plants grown under 400 (the control), 1,200, and 4,000 µmol mol^−1^ [CO_2_] reached 1.09 developmental stage (early vegetative stage) on 18, 17 and 17 DAP, and 1.14 stage (late vegetative stage) on 25, 21, and 21 DAP, respectively. The [CO_2_] influence on the rate of developmental progression was greater after stage 1.09 when there was greater leaf area to take advantage of the more abundant [CO_2_]. Our results are consistent with previous findings of field-grown Arabidopsis where plants grown at ambient or 550 µmol mol^−1^ [CO_2_] for 12 days did not exhibit different growth rates [32]. Moreover, enrichment of [CO_2_] to a super high level (4,000 µmol mol^−1^) showed no further acceleration of development beyond that at 1,200 µmol mol^−1^. Although the differential physiological responses to elevated and SE CO_2_ have been seen in both monocotyledon and dicotyledon plant species [17], our study demonstrated a similar differential response of biomass accumulation and transpiration to elevated and SE [CO_2_] in the model plant, Arabidopsis. Therefore, Arabidopsis can be used to study the molecular and biochemical basis for such physiological responses.

This study further demonstrates that many of the molecular and metabolite responses to both elevated and SE [CO_2_] are under the control of programmed plant development. On a narrow scale, in response to elevated [CO_2_], many genes up- or down-regulated at one developmental stage often exhibited a different or opposite response at another developmental stage (Table 2), this was unexpected. In particular, transcript profiles for photosynthetic acclimation in response to elevated [CO_2_] (1200 µmol mol^−1^) appears to be a strong function of developmental stage (Figure 4B). For example, there was a differential effect on the expression of genes that encode components of the photosynthetic machinery and chloroplast energy metabolism between elevated and SE [CO_2_] (Figure 4B, Table S1B). The down-regulation of expression of several genes encoding photosynthetic electron transfer components, chloroplastic ATP synthase and NADPH dehydrogenase at the early vegetative stage suggests a prominent coordinate repression for energy capture at the early vegetative stage relative to an increased expression at the late vegetative stage at elevated CO_2_ (1,200 µmol mol^−1^) (Figure 4B, Table S1B). This response is highly reminiscent of the [CO_2_]-induced acclimation response of photosynthesis during long-term exposure to elevated [CO_2_] [48]. That is, there is a reduction in photosynthesis, gene expression of components of photosynthesis and/or reduced protein levels and activity of enzymes of photosynthesis during long-term exposure to elevated [CO_2_]. This response has largely been associated with reduced activity or expression of RuBisCO [20], [48], [49], [50], [51]. In this study, at the early vegetative stage of development, the down-regulation of components of photosynthetic electron transport and energy capture as ATP and NADPH in plants grown at 1,200 µmol mol^−1^ [CO_2_] is in accord with the process of photosynthetic acclimation [32], [48]. To our surprise, the expression pattern observed at the early vegetative stage was found to be almost perfectly the opposite of the response at the late vegetative stage where the overall expression pattern was largely an up-regulation. From these data, we conclude, at the gene expression level, photosynthetic acclimation is a clear function of developmental stage. This fact of developmental control over responses to elevated [CO_2_] in terms of photosynthetic adjustments may be part of the reason why the literature contains many conflicting reports on photosynthetic acclimation [48]. These results are consistent with recent findings that developmental stage has a major influence on the gene expression responses of plants to elevated [CO_2_] [24], [30], [31].

**Table 2:** Summary of Transcriptional and Metabolic Response to Elevated and SE [CO_2_].

	1,200 vs 400 µmol mol^−1^		4,000 vs 400 µmol mol^−1^	
	1.09 stage	1.14 stage	1.09 stage	1.14 stage
Transcripts for PSI&II genes	↓	↑	–	–
Transcript for RuBisCO	↓	↑	–	–
Starch Content	↑	↑	↑	↑
Transcript for Starch Synthesis	↑	↑	↑	↑
Transcript for BAM 3 (Starch degradation)	–	↑	↑	–
Maltose	–	↑	↑	–
Respiration metabolites	↓	↓	↓	↓
TCA metabolites	–	↓	–	↓

In contrast to plants grown at elevated [CO_2_], plants grown at SE [CO_2_] did not show changes in gene expression indicative of photosynthetic acclimation. The effect of developmental progression on photosystem gene expression at 4,000 µmol mol^−1^ was found to be very comparable to that of 400 µmol mol^−1^ [CO_2_] grown plants (Figure 4B). Unlike the expression patterns for components of photosynthesis observed for plants grown at elevated [CO_2_], growth at SE [CO_2_] had a strong dampening effect on the modulation of gene expression at both developmental stages. The signaling mechanism for photosynthetic acclimation response appears to be not occurring or is not effective at SE [CO_2_]. That photosynthetic acclimation at the gene expression level failed to occur in plants grown at SE [CO_2_] provides the first molecular evidence that there is a fundamental disparity in the way plants respond to elevated and SE [CO_2_]. Given that the rate of biomass accumulation was not further increased in plants at 4,000 compared to plants at 1,200 µmol mol^−1^, the present work implies a molecular basis for such growth responses. Thus these findings indicate an upper limit of the [CO_2_] level beyond which plants cannot acclimate.

Carbon dioxide is both an environmental cue and substrate of photosynthesis. A common response of plants to elevated [CO_2_] is accumulation of starch in photosynthetic organs [6]. Consistent with field grown Arabidopsis plants [24], a positive correlation between shoot starch levels and elevated CO_2_ was observed at the early and late vegetative stage. Similar to elevated CO_2_, in response to SE CO_2_, plants accumulated higher starch at the early vegetative stage that that of late vegetative stage. However, starch accumulation was lower at the late vegetative stage in response to both CO_2_ treatments. An accelerated demand by growth may have contributed to a reduction in shoot starch at the late vegetative stage at all [CO_2_] levels (Figure 4B). However, at the early as well as the late vegetative stage, enhanced starch levels had little effect on mid-day maltose levels (Figure 4C) consistent with Kanani at al.’s [22] findings, even though the transcript levels of genes that encode for starch hydrolysis enzymes increased at the late vegetative stage. The increased growth rate likely masked increased capacity for maltose production, which suggests that the availability of assimilates was a limiting factor for plant developmental progression. Overall, the maltose response to increasing [CO_2_] corresponded to the BAM transcript profile. This might be because BAM plays a major role in starch degradation and produces maltose in Arabidopsis [52]. Similarly, glucose and fructose were markedly higher (Figure 6) at the late vegetative stage than at the early stage. The consistently higher levels of the soluble sugars at the late vegetative developmental stage not only reflects the biochemical and metabolic linkage of these metabolites, but also suggests an increased breakdown of starch due to high demand for rapid growth.

Soluble sugars, besides being metabolites of primary metabolism, have signaling roles during growth and development and carbon partitioning between source and sink tissues [53], [54], [55], [56], [57]. Also, it was reported that during elevated CO_2_ (700 µmol mol^−1^), phytohormones like indoleacetic acid, gibberellic acid (GA3), zeatin ribose (ZR), dihydrozeatin riboside (DHZR), and isopentyladenoside (IPA) were increased and ABA was decreased [58]. In the present study, total soluble sugar levels increased in response to SE CO_2_ at the early vegetative growth stage and in response to both elevated and SE CO_2_ the late vegetative stage consistent with literature where elevated CO_2_ resulted in increased soluble sugars [18], [22], [23], [24]. Many genes that are reported to be glucose inducible were increased in this data set and many JA and ABA inducible genes were also induced (Figure 7, Table S3). Even though this study cannot answers specific questions between sink and source tissues, it points to sugar signaling pathways and potential cross-talks with hormone (JA, ethylene, auxin, ABA, gibberellins, cytokinins) signaling pathways which are involved in plant growth, development, biotic and abiotic stress responses [38], [54], [59], [60], [61], [62] during adaptation to elevated and SE [CO_2_]. Arabidopsis mutants that are insensitive to sugar signaling and other plant hormones such as JA and ABA, can be used to understand how sugar signaling and plant hormone pathways cross talk during high CO_2_ adaptation. Soybean and maize mutants can be used to understand the role of sugar and hormone cross talk in sink or source leaves during adaptation to elevated and SE CO_2_. This knowledge can help to improve crop yield and production in closed environments like space habitats where CO_2_ levels can go up to 4000 µmol mol^−1^ and due to human activity and climate change where increase in temperature and [CO_2_] is expected.

While there is broad agreement that high atmospheric [CO_2_] stimulates photosynthesis in C3 plants, such as soybean, no such consensus exists on how rising CO_2_ levels will affect plant respiration. Although respiratory flux (CO_2_ efflux and O_2_ uptake) was not measured in this study, findings that steady state level of respiratory intermediates such as glycine were decreased, and TCA cycle metabolites were either unchanged or decreased in response to both CO_2_ treatment support the long-held view that elevated CO_2_ inhibits respiration and consistent with Li et al. [24], but contradict with the recent report by Leakey et al [7] that revealed an increased respiration in elevated CO_2_-grown soybean. This conclusion was based on greater abundance of more than 90 transcripts, encoding many components of starch metabolism, sugar metabolism, glycolysis, the tricarboxylic acid (TCA) cycle and mitochondrial electron transport. Furthermore, Leakey et al. [7] reported a greater amount of carbohydrate substrate available and stimulated rates of respiratory O_2_ uptake and CO_2_ release. Since we demonstrated that the plants respond to CO_2_ differently at the two growth stages, this seemingly contradictory result may be explained by the fact that our findings were based on the vegetative stage, while that of Leakey et al. [7] was at the reproductive stage (spanning from full flowering to full seed).

In conclusion, our data demonstrates that there is a complex interaction between [CO_2_] and developmental signals, and thus gene expression cannot be modeled by a single-sampling point analysis. In other words, the influence of elevated and SE [CO_2_] on gene expression is modulated in concert within the developmental context of the plant. At elevated CO_2_, plant acclimated by down regulating photosynthetic machinery gene expression at the early vegetative stage, but not late vegetative stage. In contrast to elevated CO_2_, at SE [CO_2_], plants do not seem to photosynthetically acclimate at either early or late vegetative stage at the gene expression level, suggesting that there is an upper limit to the level of [CO_2_] where plants can optimally acclimate and downward regulate the machinery of photosynthesis. Both elevated and SE [CO_2_] treatments resulted in accumulation of starch at early and late vegetative stages, but the magnitude of accumulation was modulated by development. At the early vegetative stage, elevated CO_2_ did not significantly affect soluble sugar content, but SE CO_2_ resulted in increased soluble sugar content. However, at the late vegetative stage both elevated and SE CO_2_ resulted in higher soluble sugar content. The higher levels of soluble sugars at the late vegetative stage suggest an increased breakdown of starch due to the demand of rapid growth. Furthermore, the sugar signaling and cross talk between sugar and hormonal signals may be important to improve crop yield during elevated CO_2_ and SE CO_2_ levels in closed culture vessels.

## Materials and Methods

### Plant Cultivation and Elevated [CO_2_] Treatments

*Arabidopsis thaliana* (var. Columbia) seeds were stratified at 4°C for three days and sown in autoclaved soil mix (Sunshine Mix 5, SUN GRO) through 16 holes made in the lids of 11.2 cm^2^×7.8 cm tall delicatessen containers (refer to as deli-pot hereafter, a gift from Publix Super Markets Inc., Lakeland, FL). About 2 to 3 seeds were sowed in one hole. Deli-pots were transferred to a 12 ft^2^ controlled environment chamber equipped with cool white fluorescent lamps. The photoperiod was 16/8 h light/dark cycle at 20°C±2°C with 65% relative humidity throughout the experiment. Irradiance levels were kept at 150 µmol m^−2^ s^−1^ photosynthetically active radiation (PAR) and ambient CO_2_ (400 µmol mol^−1^) for the first 10 days following sowing (germination phase). After germination, seedlings were thinned out so that there was only one plant per hole and 12 plants per deli-pot. Ten day-old Arabidopsis plants were either kept under ambient CO_2_ (experimental control) or subjected to elevated and super-elevated [CO_2_] (1,200 and 4,000 µmol mol^−1^, respectively) treatments under an increased irradiance of 400 µmol m^−2^ s^−1^ PAR for the rest of the experimental duration. Plants were fertilized with 1/4 strength Hoagland solution at 9 and 11 days after planting (DAP). Each CO_2_ treatment was replicated three times in space. That is, the growth chamber was blocked into 6 plots (each plot contains 8 pots of plants), and 2 plots serves one experimental replicate.

### Sampling for Transcript and Metabolite Analyses

Regardless of the CO_2_ treatments, samples were taken from each experiment based on the actual developmental stage instead of day after sowing to eliminate confounding effects deriving from both CO_2_ and developmental stage. That is, aerial tissues of 12–16 representative plants from each plot of individual experimental treatment were harvested in the middle of the principal growth stage 1 (1.08–1.09 leaf stage) and at the end of principal growth stage 1 (1.12–1.14 leaf stage) according to Boyes et al. [63]. Compared to soybean and maize, *Arabidopsis* has very small leaves, which are also very close to each other. The source and sink leaves were not separated to collect the samples very fast and to see the transcriptional or metabolic changes in response to CO_2_ treatments. There were three plots per experimental treatment, thus three independent experimental replicates per treatment at either harvest time. All samples were taken consistently at the mid-point of photoperiod (8 h into the light period) to avoid circadian modulation of the sampled transcripts and metabolites, and plunged in liquid nitrogen immediately. Flash-frozen samples were stored at −80°C until analysis. Frozen leaf tissues were ground to a fine powder in liquid nitrogen using a mortar and pestle, and immediately used for RNA and metabolite extraction.

### RNA Extraction and Microarray Data Collection

RNA was extracted using QIAGEN RNeasy Plant Mini Kits (QIAGEN) according to the manufacturer’s protocol. Amount of total RNA was determined using an UV spectrophotometer at 260 nm. RNA preparation of each sample was diluted to a predetermined concentration for hybridization according to the protocols outlined in the GeneChip® Expression Analysis Technical Manual (Affymetrix Santa Clara, CA). Briefly, eight µg RNA was used as template for first strand cDNA synthesis (Superscript, Invitrogen) which was primed with a T7-(dT)_24_ primer containing a T7 RNA polymerase promoter sequence (Genset Oligos). *In vitro* transcription was performed on the second strand product using biotinylated UTP and CTP (Bioarray High Yield RNA Transcript labeling Kit, Enzo Diagnostics). Biotinylated cRNA was heated in Mg buffer resulting in 35–200 base fragments. Arabidopsis arrays (Affymetrix) were hybridized for 16 h at 45°C with 15 µg of fragmented cRNA. Arrays were stained with a streptavidin-phycoerythrin conjugate (Molecular Probes) and scanned with an Agilent argon-ion laser with a 488 nm emission and a 570 nm detection (GeneArray™ Scanner). Data files for each array, containing intensity data for each probe cell, were analyzed with Probe Profiler™ (Corimbia) to generate quantitative estimates of gene expression (i.e. transcript abundance). Probe sets that did not yield signal under any experimental condition were removed from further analyses. The raw transcript data is provided as supplemental data (Table S4).

### Metabolite Extraction and Analysis by GC/MS

Samples for the metabolite extraction came from the same pool of ground Arabidopsis as samples for RNA extraction. Three analytical replicates were performed for each experimental replicate. The procedure used by Wagner et al. [64] was followed for the metabolite extraction and derivatization prior to GC/MS analysis. Aliquots of 57–64 mg frozen powder were weighed accurately in a vial and a predetermined volume of hot methanol along with a known amount of ribitol (serving as an internal standard) was added to the vial. Methanol extract was partitioned against chloroform_._ The polar fraction was dried and derivatized by methoxyamination of carbonyl moieties followed by silylation of acidic protons with N-methyl-N-(trimethylsilyl)-trifluoroacetamide (MSTFA). A mixture of n-alkanes from C_12_ to C_36_ was spiked into each sample for the determination of retention time indices (RI) to facilitate the identification of metabolites. Silylated samples were analyzed immediately in a random order by a single quadrupole GC-MS system (Model: TRACE DSQ, Thermo Finnigan Corp. Austin, TX, USA). GC/MS chromatograms of samples were collected, and analyzed qualitatively using an Automated Mass Spectral Deconvolution and Identification System (AMDIS) version 2.64 in RI calibration mode. Identification of metabolites was carried out by comparing the RIs and mass spectra of samples with those in a customized GC/MS database obtained from Max Planck Institute for Molecular Plant Physiology (Golm, Germany). Metabolites were positively assigned if their mass spectrum matching factors were greater than 800 on a scale of 0–1000 and the difference of RIs between the unknown and a known standard was within ±3.0. One or two mass fragments from each identified compound were selected as quantifiers. The peak areas of these quantifiers were integrated, normalized to plant fresh weight and to the peak area of the internal standard ribitol. The resulting normalized response values were used as an estimate of metabolite abundance. The raw metabolite data is provided as supplemental data in Table S5.

### Determination of Starch

Approximately 100 mg (wet weight) samples were extracted with 500 µl 100% ethanol and 250 µl 80% ethanol twice to remove soluble sugars and vacuum dried. Starch was solubilized in 200 µl boiling water for 30 min and centrifuged. The supernatant (25 µl) was reacted with iodine-potassium iodide (0.12%) and color intensity was measured at 620 nm using a UV/VIS spectrophotometer (Spectramax, Molecular Devices, Sunnyvale, CA). Potato starch was used as the standard to estimate starch quantity.

### Databases for Microarray Data Annotation and Metabolic Pathways

Affymetrix ATH1 chips were used for the microarray analysis. Data was annotated using Windows Microsoft Access database and according to TAIR 2007 annotations (ftp://ftp.arabidopsis.org/home/tair/Microarrays/Affymetrix/old/affy_ATH1_array_elements-2007-5-2.txt). The predicted subcellular localization of the various enzymes was indicated according to the TAIR website ftp://ftp.arabidopsis.org/home/tair/Proteins/Properties/.

Metabolic pathway analysis was carried out based on transcripts that were statistically significant using aracyc metabolic pathways. The pathways were downloaded to Microsoft access database from TAIR (ftp://ftp.arabidopsis.org/home/tair/Pathways/) and MapMan [65] version 3.0.0 from 17.06.2009 (http://www.gabipd.org/projects/MapMan/) for annotation and classification of the pathways. Only the annotations from MapMan were reported.

CO_2_-responsive transcripts (Table S1B) were examined using and Transcriptional Regulation by ABA Signaling (TRABAS http://www.bioinformatics.org/trabas/) [43] and The Bio-Array resources for Arabidopsis Functional Genomics (http://bar.utoronto.ca/affydb/cgi-bin/affy_db_exprss_browser_in.cgi?pub=&dataset=bar) for abiotic stress (temperature stress, drought, oxidative, osmotic, UV-B, genotoxic stress) and pathogen responses (*Phytophthora infestans, Botrytis cinerea, Pseudomonas syringea, Erysiphe orontii*, and elicitors derived from bacteria and oomycetes).

### Statistical Analysis

All analyses were performed using R software (http://www.r-project.org/). Principle Component Analysis (PCA) was used to analyze the gene expression and the metabolites profiling data to assess the quality of datasets and overall variability. The gene expression dataset was also analyzed using a two-way ANOVA. In addition, gene expression and metabolite profiling datasets were analyzed using a mixed effect model (http://cran.r-project.org/web/packages/nlme/index.html) to address situations where both fixed and random effects were present. Because the randomness of the samples and the machine readings represent two levels of randomness, the data structure allowed the use of the mixed effect model, which is considered to be more robust than the conventional linear models without considering the differences of randomness. The mixed effect model worked well to show [CO_2_] effects, developmental effects, and interaction of [CO_2_] with developmental stage on metabolite abundance and gene expression.

## Supporting Information

Table S1 A. Corresponding genes of the development-responsive transcripts with 2 fold or more changes in their signals in mixed effect model analysis (p-value ≤0.001). B. Corresponding genes of the CO_2_ regulated transcripts. CO_2_ only or development independent and dependent CO_2_ regulated transcripts with 2 fold or more changes in their signals in mixed effect model analysis (p-value ≤0.001) were presented.
(XLSX)

Table S2 **CO2 or developmental responsive low molecular weight compounds with 2 fold or more changes in their steady state levels in mixed effect model analysis (p-value ≤0.001).**
(XLSX)

Table S3 **CO_2_ responsive transcripts with biotic abiotic, hormone, and sugar regulation.** The transcripts in Table S2 are compared to publicly available microarray data sets.
(XLSX)

Table S4 **The raw data for transcript profiling.** n = 3 biological experiments.
(XLSX)

Table S5 **The raw data for metabolite profiling.** n = 3 biological experiments each having 3 analytical replicates
(XLSX)
